# *VGLUT2* rs2290045 genotype moderates environmental sensitivity to alcohol-related problems in three samples of youths

**DOI:** 10.1007/s00787-019-01293-w

**Published:** 2019-02-25

**Authors:** Maria Vrettou, Kent W. Nilsson, Catherine Tuvblad, Mattias Rehn, Cecilia Åslund, Anna-Karin Andershed, Åsa Wallén-Mackenzie, Henrik Andershed, Sheilagh Hodgins, Ingrid Nylander, Erika Comasco

**Affiliations:** 1 Department of Neuroscience, Science for Life Laboratory, BMC, Uppsala University, Box 593, 751 24 Uppsala, Sweden; 2 Centre for Clinical Research Västerås, Västmanland County Hospital Västerås, Uppsala University, Uppsala, Sweden; 3 School of Law, Psychology and Social Work, Örebro University, Örebro, Sweden; 4 Department of Psychology, University of Southern California, Los Angeles, USA; 5 Department of Organismal Biology, Uppsala University, Uppsala, Sweden; 6 Department of Clinical Neuroscience, Karolinska Institutet, Stockholm, Sweden; 7 Institut Universitaire en Santé Mentale de Montréal, Université de Montréal, Montreal, Canada; 8 Department of Pharmaceutical Biosciences, Uppsala University, Uppsala, Sweden

**Keywords:** Adolescents, Alcohol, Gene, Glutamate, Stress, VGLUT2

## Abstract

The importance of Vesicular Glutamate Transporter 2 (VGLUT2)-mediated neurotransmission has been highlighted in studies on addiction-related phenotypes. The single nucleotide polymorphism rs2290045 in *VGLUT2* has been associated with alcohol dependence, but it is unknown whether or how this association is affected by environmental factors. The present study determined whether the association of alcohol-related problems with the rs2290045 in the *VGLUT2* gene was modified by negative and positive environmental factors. Three samples were included: a clinical sample of 131 adolescents followed from age 17 to 22; a general population sample of 1794 young adults; and a general population sample of 1687 adolescents followed from age 14 to 17. DNA was extracted from saliva and the rs2290045 (T/C) was genotyped. Alcohol-related problems were assessed using the Alcohol Use Disorders Identification Test. Stressful life events (SLE) and parenting were assessed by questionnaires. Gene-environment interactions were investigated using a dual statistical approach. In all samples (effect sizes 0.6–6.2%), and consistent with the differential susceptibility framework, T carriers exposed to SLE reported more alcohol-related problems if they had experienced poor parenting, and lower alcohol-related problems if they had received supportive parenting. T carriers not exposed to SLE reported higher alcohol-related problems if they had received supportive parenting and lower alcohol-related problems if they had received poor parenting. Among CC carriers, alcohol-related problems did not vary as a function of negative and positive environmental factors. In conclusion, in three samples of youths, alcohol-related problems were associated with an interaction of *VGLUT2* rs2290045, SLE, and parenting.

**Electronic supplementary material:**

The online version of this article (10.1007/s00787-019-01293-w) contains supplementary material, which is available to authorized users.

## Introduction

Alcohol misuse is responsible for 5.1% of the global burden of disease [63] and can lead to alcohol use disorder (AUD), which is characterized by prolonged, compulsive and detrimental alcohol-drinking patterns, constant preoccupation with alcohol acquisition/drinking, tolerance and/or withdrawal symptoms [4]. Twin studies show that heritability of alcohol addiction ranges between 40‒60% [28]. Different developmental patterns and genes may be involved in the development of AUD during adolescence and adulthood [18]. Adolescence is a critical period in the development of AUD, as first use of alcohol commonly occurs during this period. After this early experimentation phase, individuals display more stable patterns of drinking [18].

Alcohol-related phenotypes (i.e. response to acute or chronic alcohol, withdrawal symptoms, loss of control over alcohol drinking/seeking, relapse) have consistently been associated with a dysfunctional glutamatergic system [32, 34]. The glutamatergic system mediates the reinforcing effects of alcohol through various mechanisms, one of which is the interaction with the dopaminergic system in the mesolimbic circuit [32]. Vesicular Glutamate Transporters (VGLUTs), 1‒3, package glutamate in the presynaptic vesicles [3] (referring to *Vglut/*VGLUT for mRNA/gene and protein, respectively, in rodents and *VGLUT/*VGLUT in humans). Thus, any *Vglut*/*VGLUT*-expressing neuron has the ability to package and release glutamate, rendering *VGLUT* genes optimal markers for the glutamatergic phenotype.

One of these three markers, VGLUT2, is broadly expressed in brain areas of relevance to addiction, e.g. the cerebral cortex, hippocampus, thalamus, amygdala and medulla [26, 64]. Within the midbrain, VGLUT2 is expressed in both glutamatergic and dopaminergic neurons of the ventral tegmental area (VTA), a key area mediating reward [47, 66]. Preclinical studies provide an association of VGLUT2 mRNA and protein expression with alcohol exposure pre- and post-natally [68, 69]. In rats having free access to alcohol, we recently observed lower *Vglut2* expression in the medial prefrontal cortex, a key region involved in executive functions such as decision making and processing environmental cues [65]. Further, studies of rodents have demonstrated the involvement of a VGLUT2 co-phenotype in behaviours of relevance to addiction. For example, mice lacking *Vglut2* in midbrain dopamine neurons show reduced locomotor response to acute injections of amphetamine [10] and cocaine [30], and higher cocaine self-administration and cue-induced drug seeking [2]. VGLUT2 has been shown to contribute to increased dopamine vesicular content by regulating vesicle acidification upon depolarization, further highlighting its relevance to dopaminergic neurotransmission [1]. Recently, it was demonstrated that VGLUT2 in dopamine neurons contributes to baseline AMPA/NMDA ratio in target neurons of the nucleus accumbens, a finding which suggests a role in synaptic plasticity of relevance to aspects of addiction [49]. Moreover, we have previously found that the expression profile of *Vglut2* in the VTA differed in rats who voluntary drank alcohol if they had been exposed to early-life stress [65]. This was the first evidence that the association of *Vglut2* and alcohol consumption was modified by stress.

In humans, an association of *VGLUT2* genotype with alcohol dependence was found by an exploratory, haplotype-tag Single Nucleotide Polymorphisms (SNPs) study of the three *VGLUT* genes, such that the minor allele of the SNP rs2290045 in *VGLUT2* was overrepresented (OR 1.660) in a sample of 191 women with alcohol dependence as compared to 184 healthy women [17]. This association could reflect a gene-by-environment interaction (GxE), since individuals presenting alcohol dependence typically have experienced more negative, and fewer positive, environmental factors, than their healthy peers [20]. Importantly, not only negative, but also positive environmental experiences may interact with *VGLUT2* to influence individual susceptibility to AUD [67].

Both supportive and aversive psychosocial factors have indeed been associated with risk for AUD. Adoption studies have shown that a positive rearing environment leads to lower risk of almost 50% in drug misuse among individuals with high genetic risk for addiction [41]. Furthermore, parental monitoring has been associated with later onset of alcohol misuse, lower rate of alcohol drinking escalation across time, and less-frequent intoxication among adolescents [7, 44]. On the other hand, many studies have shown that negative environmental factors are associated with AUD. For example, maltreatment in childhood, including neglect [24, 27, 56], and witnessing physical/verbal abuse between parents, [15, 60] have been associated with higher alcohol consumption in adolescents.

The “diathesis-stress” hypothesis [70] proposes that carriers of risk alleles show increased vulnerability to negative environmental factors. The “vantage sensitivity” framework suggests that responses to positive environmental factors depend on inherent characteristics [51]. The more recent ‘differential susceptibility’ theory [8], integrates both approaches, and postulates that depending on the genotype some individuals are more, and some less, susceptible to both negative and positive environmental factors. Similarly, the ‘biological sensitivity to context’ theory [11] postulates that GxE shape individuals’ environmental sensitivity over time, with some individuals having high biological reactivity to both highly stressful and highly protective environments [11]. Thus, both the differential susceptibility theory and the biological sensitivity to context theory propose that individuals differ in their sensitivity to negative and positive environmental factors [23]. GxE studies that include both stressful and enriching environmental factors are, therefore, needed to test these theories [23, 50].

Very few studies have tested three-way gene-by-environment interactions (GxExE) including both negative and positive environmental factors. One of the first such studies showed that a positive environmental factor (i.e. social support) moderated a genetic effect on depression among maltreated children [37]. Most other studies have examined associations of genes with various psychopathologies according to the differential susceptibility approach [8], including only one environmental variable at a time, either negative or positive, while recent results of meta-analyses provided evidence that genotypes increase sensitivity to both negative and positive environmental factors [6, 62].

To date, *VGLUT2* genotypes have been investigated in relation to neuropsychiatric outcomes, such as schizophrenia [55] and Parkinson disease [45]. To our knowledge, the exploratory haplotype-SNPs study, previously conducted by our group, is the only study investigating *VGLUT2* genotype in relation to AUD [17], however, the interaction of this genotype with environmental factors remains to be studied. Hence, to further understanding of the role of *VGLUT2*, and specifically of the rs2290045 genotype, in alcohol misuse, the present study sought to determine whether alcohol-related problems (i.e. hazardous alcohol use, dependence symptoms and harmful alcohol use [5]) were associated with interactions of *VGLUT2* SNP rs2290045 and positive and negative environmental factors. Considering the strong associations between smoking and alcohol misuse [28], and that higher *VGLUT2* gene expression has been found post-mortem in the VTA of alcoholic smokers compared to controls, and to alcoholic non-smokers [25], the potential confounding effects of nicotine use were estimated. The study focused on adolescence/young adulthood, a transitional period characterized by dramatic physical and emotional changes, novelty-seeking and risk-taking behaviors, in an attempt to identify susceptible individuals early in time.

One clinical sample and two general population samples of adolescents and young adults were studied. Guided by the environmental sensitivity framework [50], we hypothesized that individuals carrying the T allele who were exposed to stressful life events (SLE) would present more alcohol-related problems if they received non-optimal parenting, and fewer alcohol-related problems if they experienced warm, positive parenting. Among T carriers, those not exposed to SLE were expected to display fewer alcohol-related problems than those who experienced SLE, and fewer alcohol-related problems were expected with increasing quality of parenting. By contrast, it was hypothesized that alcohol-related problems would not be associated with the interaction between positive or negative environmental factors among individuals carrying CC genotypes.

## Materials and methods

### Populations and study designs

#### Clinical sample (CS)

The clinical sample (CS) included individuals who as adolescents had sought treatment for substance misuse and who were assessed when they initially (baseline) contacted a clinic in Stockholm and then 6, 12, and 60 months later. At each assessment, structured, diagnostic interviews were conducted by trained psychologists and at the 60th month follow-up, a saliva sample was collected for DNA extraction. At baseline, of the 373 individuals invited to participate, 48% agreed, and 82% of these individuals participated in the follow-up data collections [31]. Attrition rate was low (4.6%), previous analyses comparing the 61 individuals who agreed with the 61 individuals who refused to participate in the study, indicated that the sample was representative of the clinic population [31]. The final sample included 131 individuals with complete data at baseline and 125 individuals at the 60th month follow-up. Consent was obtained from each participant. The study was approved by the Karolinska Institute Research Ethics Committee Nord (Dnr03-543), and the Regional Board for Research Ethics in Stockholm, Sweden (Dnr2008/1934-31/3) [31].

#### General population sample of young adults (GP-adults)

The Retrospective Study of Young People’s Experiences (RESUMÉ) includes 2500 individuals, randomly selected from the Swedish population born between 1987 and 1991. Participants were recruited from a pool of 20,827 individuals, who had been drawn from a national population register, until the target number of 2500 had been reached. RESUMÉ examines the association of adverse and stressful experiences in childhood and adolescence with various outcomes in young adulthood including mental and physical health. About 25% of the initial population did not provide saliva sample [14]; from the remaining 75%, who provided saliva (*n* = 1870), genotyping failure accounted for 3%. In the current study, 1756 participants with complete data from questionnaires and DNA isolated from saliva were considered. Informed consent was obtained from each participant. Participants received a small monetary compensation for their participation. The study was approved by the Regional Ethics Board in Uppsala, Sweden (Dnr2010/463) [14].

#### General population sample of adolescents (GP-adolescents)

The Survey of Adolescent Life in Västmanland (SALVe) cohort includes adolescents born in 1997 and 1999 and their parents, living in the county of Västmanland, Sweden. The adolescents were contacted by mail and invited to participate in the study, which included completing questionnaires about socio-demographic characteristics and mental health, and providing a saliva sample for DNA extraction. At baseline, of the 4,875 individuals invited to participate [61], 38% agreed, and 84% of these participated in the follow-up data collection. Attrition rate was 14.9%, but did not affect genotypic frequencies, nor differences in SLE, parenting and AUDIT-C were observed between the dropouts and the participants at follow-up. The final sample included 1687 participants at baseline and 1436 at the 3-year follow-up for whom all data were available. Informed consent to participate was collected from both the adolescents and their parents. Participants received movie tickets as compensation for their time and inconvenience. The study was approved by the Regional Ethics Board in Uppsala, Sweden (Dnr2012/187).

### Assessment instruments

#### Alcohol consumption and alcohol-related problems

The Alcohol Use Disorders Identification Test (AUDIT) [54] (CS and GP-Adults) was used to measure alcohol-related problems, and the AUDIT-Consumption (AUDIT-C) [12] (GP-Adolescents) was used to measure alcohol consumption. In GP-Adolescents, a modified version of AUDIT-C was used, designed for adolescents, containing more response options for questions 1 and 2 (the response “monthly or less” was divided into “every other month or less” and “about once a month”). Nicotine use was defined as current smoking and/or use of Swedish snus in the CS and GP-Adolescents, and current smoking only in GP-Adults, where data for Swedish snus were not available. The variable was dichotomized as no use vs. occasional/daily use.

#### Environmental factors

CS participants self-reported exposure to physical abuse by parents, sexual abuse, and victimization by others during adolescence at baseline [31] and at 60th month follow-up. Physical abuse by parents was assessed, at baseline and follow-up, using the Conflict Tactics Scale Parent Child Version [59], and was defined as present if one of the following had occurred: hit with fist or kicked hard, hit with a hard object on any body part except bottoms, choked, burned, thrown/knocked down, threatened with knife/gun, beaten up [31]. Events such as slapped on the hand/arm/leg, pinched, spanked on the buttocks were considered of no/minor valence and thus classified as minor abuse. Sexual abuse at baseline was defined as affirmative report either of parents or the participant in the Sexual Experience Survey questionnaire [36]. Sexual abuse at follow-up was assessed using four items from the Sexual and Physical Abuse Questionnaire [42], or one item (“Has anyone physically forced you to have sex against your will?”) from the McArthur Community Violence Instrument [58], and was defined by an affirmative response to any of the questions. Victimization by peers was assessed at baseline using a self-report questionnaire of 7 items regarding victimization during the past 6 months with the responses ranging from 0 (no victimization) to 7 (affirmative response in 7 items) and was defined by an affirmative response [31]. Victimization by others was assessed at follow-up using 8 items from the McArthur Community Violence Instrument [58] regarding exposure to aggressive behaviour with responses ranging from 0 (no victimization) to 8 (affirmative response in 8 items) and was defined by an affirmative response. In the current study, each variable was dichotomized as 0 (no/minor) or 1 (high). A combined variable indexing SLE was created for each time-point (baseline and follow-up) considering all three types of maltreatment and victimization, ranging from 0 (no/minor) to 3 (3 types of SLE). Regarding parenting, a factor analysis was performed including 58 self-reported items, rating the relation between the participants and their parents from poor to good (0–4). The three most important dimensions were: child-parent openness, parent–child affect, and support. For each dimension, a summation index was computed ranging from 0 to 24 (openness, affect) and 0 to 16 (support); higher scores indicated higher levels of positive parent–child relationship (for a more detailed description see Table S4).

##### GP-adults

Participants self-reported lifetime exposure to physical violence (11 items), verbal aggression (2 items), sexual abuse (7 items), neglect (5 items), and witnessing violence (7 items), on a response scale ranging from 0 (none) to 5 (five or more times) [14]. For each variable, SLE was defined as events that had occurred twice or more [14]. A combined SLE variable was created taking account of all types of SLE, ranging from 0 (no/minor) to 5 (5 types of SLE). The quality of the parent–child relationship from birth to age 18 was assessed using two questions regarding the relationship of the participant with their mother or father rated on a scale from 0 (poor) to 3 (supportive). The answers were used to create a summation index ranging from 0 to 6; with higher score indicating higher level of supportive parenting (for a more detailed description see Table S4).

##### GP-adolescents

Witnessing physical and/or verbal abuse between parents, and from the parents towards the participant was assessed using four questions. Each question was rated by the adolescents on a scale from 0 (No) to 5 (Yes, every/almost every day). Scores were summed to create a score ranging from 0 to 20; with higher score indicating higher levels of SLE [33]. Parenting style was assessed using the Parents as Social Context Questionnaire (PASCQ) completed by the adolescents [57]. A positive parenting index was computed, including dimensions of warmth, structure and autonomy; ranging from 3 to 36, with higher score indicating higher levels of supportive parenting (for a more detailed description see Table S4).

#### Genotype analyses

DNA was extracted from 200 µl of saliva collected with the Oragene self-collection kit (DNA Genotek^®^, Canada) using the silica-based Kleargene DNA extraction method. Genotyping analyses of the SNP rs2290045 were performed using the Kbioscience Allele-Specific Polymorphism assay based on competitive allele-specific PCR and bi-allelic scoring (LGC^®^, England). No-template control samples were included to enable the detection of contamination or non-specific amplification. Deviation from Hardy–Weinberg Equilibrium was observed in GP-Adult males, who were thus excluded from further analyses (Table S1).

### Data analysis

Genotypes were grouped as homozygous and heterozygous of the rs2290045 minor T allele and homozygous for the major allele to address the statistical constraint imposed by the low frequency of the minor allele (Table S1). Changes in alcohol-related problems over time in GP-Adolescents and the CS were tested using the non-parametric Wilcoxon signed-rank test, and bivariate correlations were tested computing the Spearman correlation coefficient (Table S3). Group differences were tested using the Pearson Chi square for categorical data and the Mann–Whitney *U* test for continuous variables (Table S3). Regarding ethnicity (Table S1), information was not available for GP-Adults while genotypic frequencies in the CS and GP-Adolescents were similar to the ones reported in public databases for Caucasians.

**Fig. 1:**
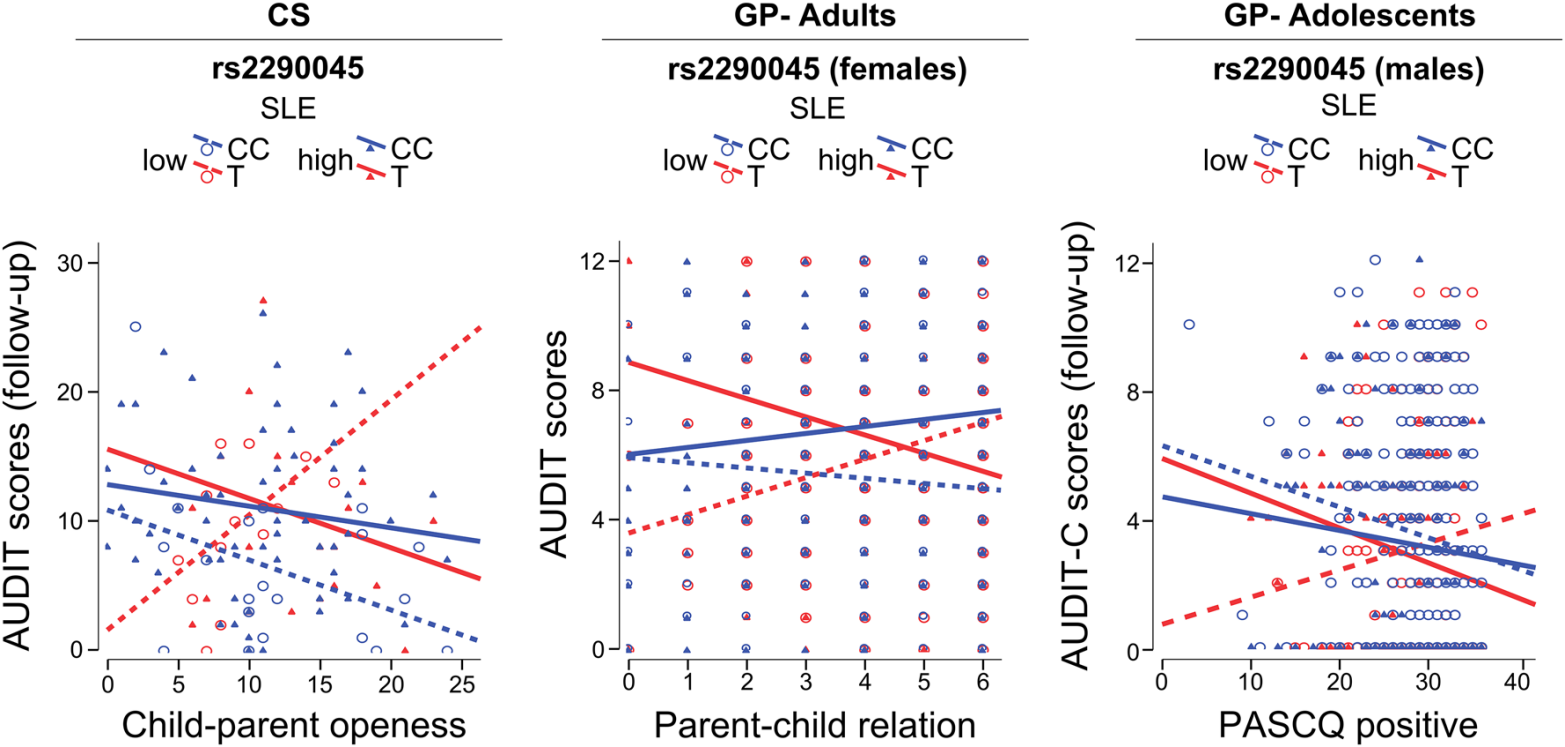
Fit regression lines depicting the interaction between negative and positive environmental factors, on alcohol-related problems in rs2290045 T and CC carriers. Dichotomization of the SLE variable was performed only to illustrate the interaction as follows: clinical sample (CS): low SLE: < 1; high SLE: ≥ 1; general population (GP)-adults: low SLE: ≤ 1; high SLE: ≥ 2; general population (GP)-adolescents: low SLE: < 1.1; high SLE: ≥ 1.1. AUDIT: Alcohol Use Disorders Identification Test; AUDIT-C: AUDIT-Consumption; PASCQ: Parents as Social Context Questionnaire

Multivariable statistical modelling according to *Keller* [39] was used to examine associations of three-way interactions of genotype and positive and negative environmental factors and AUDIT/AUDIT-C scores (Table S2). Two approaches were used; one parametric (univariate General Linear Model (GLM) test, two-way ANOVA with Type III sum of squares), and one non-parametric (Negative Binomial (NB) Generalized Linear Model with robust estimator covariance matrix and maximum likelihood estimation). Sex was entered into models as a covariate, except in analyses of GP-Adults that included only females, and the sex-by-genotype and sex-by-environment terms were included in the statistical model as suggested by [39] (Table S2). When the three-way interaction of interest was not statistically significant, sex was included in the interaction term (4-way interaction term) to test for interactions modified by sex; when significant 4-way interactions were observed, the relationship was further investigated separately by sex (Table S2). Nicotine use was taken into account as a potential confounder in separate analyses (Table S2). The partial eta square was used as indicator of the effect size. The combined SLE variable in each sample was dichotomized to illustrate the interaction terms (Fig. 1). All interactions were further probed using PROCESS macro in SPSS [29] and the regions of significance (ROS) were identified [52].

## Results

### Descriptive characteristics

Characteristics of the three samples are presented in Table 1, and by sex in Table S3. Among GP-Adolescents, but not in the CS, alcohol consumption differed over time (*Z* = − 25.304, *p* < 0.0001). Only 12% (*N* = 201) of GP-Adolescents were consuming alcohol when they were, on average, 14 years old. Differences in AUDIT/AUDIT-C scores depending on sex, genotype, SLE and parenting are presented in Table S3. Aggregation of the three samples showed no main effect of genotype on AUDIT-C scores. Weak correlations were observed between AUDIT/AUDIT-C scores and environmental variables (Table S3), whereas no gene-environment correlation was found.

**Table 1:** Descriptive characteristics in the clinical sample (CS), general population (GP)-adults and -adolescents

CS			GP-adults		GP-adolescents		
Variable	Mean ± SD, range (%)		Variable	Mean ± SD, range (%) (*N* = 1756)	Variable	Mean ± SD, range (%)	
	Baseline (*N* = 131)	Follow-up (*N* = 125)				Baseline (*N* = 1687)	Follow-up (*N* = 1436)
Age (years)	16.5 ± 1.85 12–20	22.2 ± 1.8 19–26	Age (years)	22.15 ± 1.4 20–24	Age (years)	14.4 ± 1.04 13–16	17.3 ± 1.04 16–19
Sex (females)	76 (58)	72 (58)	Sex (females)	927 (52.8)	Sex (females)	949 (56.3)	846 (58.9)
rs2290045 MAF	16.4	16	rs2290045 MAF	14.9	rs2290045 MAF	17.1	17
AUDIT	10.76 ± 8.16 0–40 (94)	10.02 ± 6.94 0–35 (93)	AUDIT	6.77 ± 4.8 0–30 (91)	AUDIT-C	0.42 ± 1.39 0–11 (12)	3.3 ± 3.3 0–14 (63)
SLE (types)			SLE (types)		SLE		
None	26.7%	29.6%	None	30.6%		0.78 ± 1.65	1.31 ± 1.94
One	35.1%	35.2%	One	29.4%		0–13 (30)	0–12 (48)
Two	27.5%	24%	Two	21.4%			
Three	10.7%	11.2%	Three	11.6%			
			Four	5.2%			
			Five	1.8%			
Child-parent openness	11.32 ± 5.72 0–24		Parent–child relationship	4.32 ± 1.59 0–6	PASCQ positive		28.3 ± 5.3 3–36
Parent–child affect	11.78 ± 5.9 0–24						
Parent–child support	10.25 ± 3.72 2.7–16						

The *N*s refer to the individuals for whom data for all the variables used in the present study were available

*AUDIT* Alcohol Use Disorders Identification Test, *AUDIT*-*C* AUDIT-Consumption, *MAF* minor allele frequency, *PASCQ* Parents as Social Context Questionnaire, *SLE* stressful life events

### Associations of alcohol-related problems and interactions of rs2290045, maltreatment, and parenting

In the CS, AUDIT scores at follow-up were associated with an interaction of rs2290045, SLE, and child–parent openness (AUDIT: GLM: (*F*_(1,107)_ = 7.018, *η*_p_^2^ = 0.062, *p* = 0.009; adj. *R*^2^ = 0.205; NB: Wald *χ*^2^ = 17.246, *p* = 0.00003). T carriers who had experienced higher levels of SLE reported higher AUDIT scores than CC carriers if they had also experienced poor child–parent openness, and lower AUDIT scores if they had enjoyed a supportive, open, relationship with parents. The opposite pattern was seen in the absence of SLE. ROS analysis showed that the interaction was significant when parenting was higher than 13.6 (range; mean ± SD: 0–24; 11.32 ± 5.72).

Among adult females in the general population sample, AUDIT scores were associated with an interaction between rs2290045, SLE, and quality of the parent–child relationship (GLM: (*F*_(1,919)_ = 9.404, *η*_p_^2^ = 0.01, *p* = 0.002; adj. *R*^2^ = 0.030; NB: Wald *χ*^2^ = 9.121, *p* = 0.003). T carriers who had experienced higher levels of SLE reported higher AUDIT scores than the CC group if they had also experienced poor parenting, but lower AUDIT scores if they had enjoyed supportive parenting. The opposite pattern was seen in the presence of lower levels of SLE. ROS analysis showed that the interaction was significant when parenting was lower than 1 and higher than 4.4 (range; mean ± SD: 0–6; 4.32 ± 1.59).

Among the adolescents in the general population sample, follow-up AUDIT-C scores were borderline significantly associated with a four-way interaction between rs2290045, SLE (follow-up), parenting style and sex (GLM: *F*_(1,1415)_ = 3.063, *η*_p_^2^ = 0.002, *p* = 0.08, adj. *R*^2^ = 0.030; NB: Wald *χ*^2^ = 3.152, *p* = 0.076). The model was re-run separately among males and females. Among males, AUDIT-C scores were associated with a three-way interaction of genotype, SLE, and parenting style (GLM: *F*_(1,581)_ = 3.754, *η*_p_^2^ = 0.006, *p* = 0.053, adj. *R*^2^ = 0.009; NB: Wald *χ*^2^ = 4.485, *p *= 0.034). T carriers who had been exposed to higher levels of SLE (follow-up), reported higher AUDIT-C scores, than the CC group, if they experienced poor parenting, but lower AUDIT-C scores if they experienced positive parenting. The opposite pattern was seen in T carriers exposed to lower levels of SLE. ROS analysis showed that the interaction was significant when parenting was between 3 and 17.5 (range; mean ± SD: 3–36; 28.3 ± 5.3).

As illustrated in Fig. 1, consistent with the environmental sensitivity framework [50], in all three samples the association of *VGLUT2* SNP rs2290045 with AUDIT/AUDIT-C scores was modified by negative and positive environmental factors. T carriers who had experienced SLE reported more alcohol-related problems if they had a poor relationship with parents, and fewer alcohol-related problems if they enjoyed a positive relationship with parents. T carriers who had not experienced SLE, reported few alcohol-related problems if they had a poor relationship with parents, and more alcohol-related problems if they had a positive relationship with parents. By contrast, among CC carriers, levels of alcohol-related problems did not differ as a function of SLE or quality of the parent–child relationship. The results were virtually similar when considering TT carriers as a separate group (data not shown).

In a separate analysis, nicotine use was considered as potential confounding factor (Table S2) and was included in the model following the abovementioned approach by *Keller* [39]. In GP-Adults, the association of AUDIT scores with the interaction of rs2290045, SLE and parent–child relationship was strengthened when nicotine use was entered in model (GLM: *F*_(1,912)_ = 12.144, *η*_p_^2^ = 0.013, *p *= 0.001; adj. *R*^2^ = 0.119; NB: Wald *χ*^2^ = 11.783, *p *= 0.001). ROS analysis showed that the interaction was significant when parenting was lower than 1.4 and higher than 4.6 (range; mean ± SD: 0–6; 4.32 ± 1.59). In GP-Adolescents, adding nicotine use to the model, weakened the association of AUDIT scores with the interaction of genotype, SLE, and parenting style (rs2290045 ×  SLE (follow-up) × PASCQ positive on AUDIT scores (follow-up): GLM: *F*_(1,574)_ = 2.823, *η*_p_^2^ = 0.005, *p* = 0.093, adj. *R*^2^ = 0.267; NB: Wald *χ*^2^ = 2.345, *p* = 0.126), as well as in the CS, (rs2290045 × SLE (follow-up) × child-parent openness on AUDIT scores (follow-up): GLM: *F*_(1,94)_ = 6.907, *η*_p_^2^ = 0.068, *p* = 0.01; adj. *R*^2^ = 0.174; NB: Wald *χ*^2^ = 13.645, *p* = 0.00002). ROS analysis showed that the interaction was significant when parenting was between 3 and 20.5 (range; mean ± SD: 3–36; 28.3 ± 5.3) for GP-Adolescents, and when it was lower than 3 and higher than 15.1 (range; mean ± SD: 0–24; 11.32 ± 5.72) for the CS.

## Discussion

### Main findings

The present study sought to determine whether alcohol-related problems were associated with interactions of *VGLUT2* SNP rs2290045 and positive and negative environmental factors in one clinical sample and two general population samples of adolescents and young adults. The main finding was that T carriers differ in their alcohol-related problems depending on their environmental exposure, a plasticity not seen in CC individuals.

Consistent with the environmental sensitivity theory [50], and in line with the overrepresentation of the minor allele (T) of rs2290045 among individuals with AUD [17], in all three samples we found that T carriers, but not CC carriers, reported more alcohol-related problems if they had experienced SLE and poor parenting (effect sizes 0.6–6.2%). Among T carriers, but not CC carriers, who were not exposed to SLE, there was a positive association between alcohol-related problems and positive parenting. Thus, our hypothesis that T carriers raised in supportive environments would report fewer alcohol-related problems was confirmed among those who had experienced maltreatment. It may be that T carriers who experienced SLE and poor parenting drank excessively as a strategy to cope with stress [16]. The finding that T carriers who were not maltreated and who received high quality parenting reported high levels of alcohol-related problems was unexpected. One interpretation of this finding is that T carriers adapt well to their environments [8, 23] for example drinking heavily in adolescence and early adulthood is typical and, in Sweden, an integral part of an active and social life [16]. Whether these associations will persist later in life and result in AUD needs to be investigated in further follow-up studies. We hypothesize that “protected” individuals, that is those who experienced neither SLE nor poor parenting, will reduce their alcohol consumption following these adolescent years of experimentation, while individuals who experienced both SLE and poor parenting will develop AUD. T carriers who had experienced SLE and positive parenting presented fewer alcohol-related problems, even fewer than CC carriers. Among CC carriers, alcohol related problems, as hypothesized, were not associated with the interaction of SLE and the quality of parenting as for T carriers, but neither did they remain totally unaffected by the environment, as they reported average or lower AUDIT scores as the quality of parenting increased. This finding suggests that among carriers of specific genotypes positive parenting mitigates the effects of negative environmental factors, as has previously been shown [9]. Importantly though, when child-genotype x parental interactions are being assessed, as in the present study, possible confounding factors of GxE effects such as the common parent/child environment should be considered as well, in an attempt of fitting GxE in a broad developmental framework [46]. Hence, further study of such interactions is needed to inform individualized prevention and treatment strategies.

The present study did not aim to examine a link between alcohol-related problems and rs2290045 but to examine the association of alcohol-related problems and the interaction of this SNP with positive and negative environmental factors. In the presence of different effects of the plasticity allele depending on the combination of positive and negative environmental factors, as found in the present study, it could indeed be questioned if there is any reason to investigate the direct effect of a plasticity allele in relation to a specific phenotype since no individual lives in a vacuum [48]. Likewise, it could also be questioned if it is meaningful to investigate just the interaction between a genotype and a negative environmental factor, since two individuals may vary regarding positive environmental factors [48]. It is essential to take account simultaneously of both negative and positive factors that exist in the real world and interact to affect outcomes. Therefore, guided by the DST/environmental sensitivity framework., two environmental factors were considered to test the GxExE, an index of stressful life events and a measure of parenting; thus allowing to investigate the interactive effect of *VGLUT2* genotype by maltreatment across a continuum from poor to positive parenting. Further, the study included two large population-based samples that, as would be expected, included only small numbers of individuals who showed high levels of alcohol-related problems, and thus it is not surprising that the direct association of rs2290045 and AUD that we previously reported in a clinical sample [17] was not replicated here.

Results of the present study were similar in males and females. In GP-Adolescents, associations of alcohol-related problems and the interaction of genotype, SLE, and parenting were similar in both sexes, although the association was only statistically significant among males. In the CS, this association was observed for the entire sample, suggesting that there were no sex-specific effects. Notably, these interactions were detected only at follow-up in the CS, though similar trends were observed at baseline. These findings are consistent with previous studies showing that environments with lower social control (i.e., at follow-up, when individuals are older) and/or with easier access to alcohol allow heighten expression of genetic effects [19, 67].

The present findings suggest that the T allele of SNP rs2290045 confers increased sensitivity to negative and positive environmental factors. In the CS, treatment-as-usual in Sweden was found not to be effective in reducing alcohol consumption [31]. The current findings hint that T carriers would be more responsive to prevention and to treatment than CC carriers. The gene coding VGLUT2 (*SLC17A6*) belongs to the solute carrier family 17 and is mapped onto chromosome 11p14. Loci on the chromosome 11 have been previously linked to AUD [22]. The SNP rs2290045 is located in an intronic region and evidence for a functional role has yet to be shown (Supplementary Material). Functionally, allelic variation in this or linked *VGLUT2* polymorphisms could influence susceptibility to context through differences in behavioural sensitization, reinforcement, craving [35], all behaviours linked to the glutamatergic and mesolimbic dopamine systems and to various phases of addiction.

### Limitation and strengths

One limitation of the study is the age, 14 years, of the adolescent sample, perhaps too young to expect alcohol-related problems. In this sample, the restricted variability in AUDIT-C scores and the small number of adolescents drinking alcohol likely reduced statistical power and may explain the borderline significance and lower effect size that was detected relative to the other samples. On the first hand, the interpretation of a small effect size, when derived from a minor allele, in a GxExE model, can be uncertain. However, the effect size for the genetically and environmentally affected individuals could be considerably higher than what suggested from the overall effect size in the total sample [48]. On the other hand, the direction of the effect observed for the adolescent sample was the same as in the other samples. A further strength of the present study was the use of two different statistical approaches to test GxExE. As well, each of the interaction terms was probed [29]. The pattern of the ROS at both extremes of the measure of parenting in the CS and GP-Adults hints towards differential susceptibility [52], while the small numbers of individuals reporting high AUDIT-C scores in GP-Adolescents could explain why the ROS was detected only in the presence of poor parenting. Quality of parenting also varied across the three samples; high supportiveness was reported by GP-Adolescents and Adults, whereas in the CS the quality of parenting was moderate, indicating exposure to less supportive family environments. Notably, only child-parent openness interacted with genotype and SLE in the CS. This suggests that adolescents’ perception of their relationship with their parents (i.e. to be open and trustworthy, to be able to share feelings and thoughts) may be more relevant than perceived affection or support (e.g. whether the parents showed warmth, love and support towards the adolescent) in the context of *VGLUT2*-by-environment interactions that are associated with alcohol consumption. The importance of the adolescent’s perception of parenting has also been highlighted by other studies; parental monitoring and communication have been found as important aspects of parenting in regards to reduced adolescent alcohol drinking [13, 53].

It is well known that psychosocial factors, as the ones considered in the present study, influence predisposition to alcohol use and misuse [20]. As expected, the correlations between AUDIT/AUDIT-C scores and environmental variables were weak. These correlations can be seen as necessary in a moderation analysis approach, where the relationship between the environment and the outcome differs depending on the moderator (here genotype) [43].

The principal strength of the study was the inclusion of three independent samples, of different ages (from adolescence to young adulthood) and alcohol-drinking patterns, which could be seen as a novel finding supported by two replications in independent samples that is strongly indicative of the robustness of the findings [21]. Another important strength of the study was that both negative and positive environmental factors were considered, as environmental sensitivity models suggest [50], an approach rarely seen in the literature [37, 38]. An additional strength was the use of validated psychometric tools to measure the environmental factors, although retrospective self-reports may be influenced by recall or response bias. Attrition, a potential confounding factor in longitudinal and large studies, was considered in each sample; however, in the current study the interaction was present, and the direction of the effect was the same, in all three samples, implying random missingness of data. Despite slightly different definitions of SLE across the samples, results of the interactions with the SNP and with parenting were similar. The AUDIT was used in GP-Adults and the CS to cover the whole spectrum of alcohol-related problems, while AUDIT-C was used in the younger GP-Adolescent sample to assess alcohol consumption. Nonetheless, high AUDIT-C scores indicate alcohol-misuse, a precursor of alcohol-related problems [40]. Despite these differences in the measures used in the three samples, similar findings were observed and thereby strengthening confidence in the validity of the results [21]. The associations of alcohol-related problems with interactions of rs2290045, SLE, and parenting, were not confounded by nicotine use. Moreover, a dual statistical approach was followed, using both parametric and non-parametric statistics due to the skewed nature of the outcome variable. Similar results were obtained thus confirming the robustness of the findings; however, independent replication in larger samples is needed.

## Conclusion

In three independent samples, alcohol-related problems were associated with an interaction of *VGLUT2* rs2290045 genotype, SLE, and parenting. Results suggest that the T allele increases sensitivity to the environment and that carriers of this specific allele would be most responsive to prevention and treatment programs aimed at reducing alcohol use.

## Electronic supplementary material

Below is the link to the electronic supplementary material.

Supplementary material 1 (DOCX 51 kb)
