# Supplementary material for: VGLUT2 rs2290045 genotype moderates environmental sensitivity to alcohol-related problems in three samples of youths
Source: Eur Child Adolesc Psychiatry. 2019 Feb 25;28(10):1329–40. doi: 10.1007/s00787-019-01293-w (PMC6785645; doi:10.1007/s00787-019-01293-w)
Supplement: Supplementary file 1 — Supplementary material 1 (DOCX 51 kb) [file 787_2019_1293_MOESM1_ESM.docx]

*European Child & Adolescent Psychiatry*

***VGLUT2* rs2290045 genotype moderates environmental sensitivity to alcohol-related problems in three samples of youths**

Maria Vrettou^a^, Kent W Nilsson^b^, Catherine Tuvblad^c, d^, Mattias Rehn^b^, Cecilia Åslund^b^, Anna-Karin Andershed^c^, Åsa Wallén-Mackenzie^e^, Henrik Andershed^c^, Sheilagh Hodgins^f^, Ingrid Nylander^g^, Erika Comasco^a*^

1. Department of Neuroscience, Science for Life Laboratory, Uppsala University, Uppsala, Sweden
2. Centre for Clinical Research Västerås, Uppsala University, Västmanland County Hospital Västerås, Sweden
3. School of Law, Psychology and Social Work, Örebro University, Sweden
4. Department of Psychology, University of Southern California, Los Angeles, USA
5. Department of Organismal Biology, Uppsala University, Uppsala, Sweden
6. Department of Clinical Neuroscience, Karolinska Institutet, Stockholm, Sweden and Institut Universitaire en Santé Mentale de Montréal, Université de Montréal, Canada
7. Department of Pharmaceutical Biosciences, Uppsala University, Uppsala, Sweden

* Corresponding authors:

Dr. Erika Comasco, Department of Neuroscience, Science for Life Laboratory, Uppsala University, BMC, Box 593, 751 24 Uppsala, Sweden; telephone + 46 18 471 50 20; email: [erika.comasco@neuro.uu.se](mailto:erika.comasco@neuro.uu.se)

**Supplementary Material**

**Supplementary Tables**

**Table S1.** Genotype frequencies by sex in the three samples

| **Sample**  **SNP ID** | **CS** | | | | **GP-Adults** | | **GP-Adolescents** | | | |
| --- | --- | --- | --- | --- | --- | --- | --- | --- | --- | --- |
| **rs2290045** | **Baseline**  (N = 131) | | **Follow-up**  (N = 125) | | (N = 1756) | | **Baseline**  (N = 1687) | | **Follow-up**  (N = 1436) | |
|  | **Males**  (N = 55) | **Females**  (N = 76) | **Males**  (N = 53) | **Females**  (N = 72) | **Males**  (N = 829) | **Females**  (N = 927) | **Males**  (N = 738) | **Females**  (N = 949) | **Males**  (N = 590) | **Females**  (N = 846) |
| **MAF** | 16.4% | 16.4% | 15.1% | 16,74% | 15.6%^#^ | 14.3% | 16.9% | 17.3% | 16.6% | 17.3% |
|  | **N (%)** | **N (%)** | **N (%)** | **N (%)** | **N (%)** | **N (%)** | **N (%)** | **N (%)** | **N (%)** | **N (%)** |
| CC | 38 (69.1) | 52 (68.4) | 38 (71.7) | 49 (68.1) | 601 (72.5) | 684 (73.8) | 514 (69.6) | 650 (68.5) | 411 (69.7) | 580 (68.6) |
| TC | 16 (29.1) | 23 (30.3) | 14 (26.4) | 22 (30.6) | 198 (23.9) | 221 (23.8) | 199 (27) | 269 (28.3) | 162 (27.5) | 240 (28.4) |
| TT | 1 (1.8) | 1 (1.3) | 1 (1.9) | 1 (1.4) | 30 (3.6) | 22 (2.4) | 25 (3.4) | 30 (3.2) | 17 (2.9) | 26 (3.1) |

CS: Clinical Sample; GP: General Population; MAF: minor allele frequency; ^#^ not in Hardy-Weinberg Equilibrium

No sex differences in genotype frequencies were found in any sample. Information about ethnicity was not available for GP-Adults. In GP-Adolescents and CS, self-reported data regarding the country of birth of the participants and their parents were used as a proxy for ethnicity, and no difference was found in genotypic frequencies between the groups (i.e. individuals born in Sweden/Nordic countries who were a majority vs. rest). Frequencies were similar to the ones reported in public databases for Caucasians, who were majority.

**Table S2.** Insertion of the variables in the statistical model in the clinical sample (CS) and in the general population (GP) sample of Adults and Adolescents

|  |  | **CS** | **GP-Adults** | **GP-Adolescents** |
| --- | --- | --- | --- | --- |
| **Variables** | Dependent | AUDIT | AUDIT | AUDIT-C |
|  | Fixed factor (G) | rs2290045  (0: CC; 1: TC/TT) | rs2290045  (0: CC; 1: TC/TT) | rs2290045  (0: CC; 1: TC/TT) |
|  | Covariates | SLE (E1) | SLE (E1) | SLE (E1) |
|  |  | Child-parent openness (E2) | Parent-child relationship (E2) | PASCQ positive (E2) |
|  |  | Sex (S) |  | Sex (S) |
|  |  | Nicotine use (N) | Nicotine use (N) | Nicotine use (N) |
| **Models*** | 3-way | G; E1; E2; S;  G x E1; G x E2; G x S;  E1 x E2; E1 x S; E2 x S;  G x E1 x S; G x E2 x S;  E1 x E2 x S; **G x E1 x E2** | (Only females):  G; E1; E2;  G x E1; G x E2; E1 x E2; **G x E1 x E2** | (Separately by sex):  G; E1; E2;  G x E1; G x E2; E1 x E2;  **G x E1 x E2** |
|  | 4-way^#^ |  |  | G; E1; E2; S;  G x E1; G x E2; G x S; E1 x E2; E1 x S; E2 x S;  G x E1 x E2; G x E1 x S;  G x E2 x S; E1 x E2 x S;  **G x E1 x E2 x S** |
| **Model* considering nicotine use** | 3-way | G; E1; E2; S; N;  G x E1; G x E2; G x S;  G x N; E1 x E2; E1 x S;  E1 x N; E2 x S; E2 x N;  S x N;  G x E1 x S;  G x E1 x N; G x E2 x S;  G x E2 x N; E1 x E2 x S; E1 x E2 x N; G x S x N;  E1 x S x N; E2 x S x N;  **G x E1 x E2** | (Only females):  G; E1; E2; N;  G x E1; G x E2; G x N;  E1 x E2; E1 x N; E2 x N; G x E1 x N; G x E2 x N; E1 x E2 x N; **G x E1 x E2** | G; E1; E2; N;  G x E1; G x E2; G x N;  E1 x E2; E1 x N; E2 x N;  G x E1 x N; G x E2 x N;  E1 x E2 x N; **G x E1 x E2** |

* All the variables were inserted in the model as main effects and in all possible 2- and 3-way interactions as suggested by *Keller* [[1](#_ENREF_1)].

**#** When the three-way interaction of interest was not statistically significant, sex was included in the interaction term (4-way interaction term) to test for interactions modified by sex; when significant 4-way interactions were observed, the relationship was further investigated separately by sex

**Table S3**

**Characteristics of males and females in the Clinical Sample (CS), and in the general population (GP)-Adults and -Adolescents**

|  | **CS**  **Mean ± SD (%)** | | | |  | **GP-Adults**  **Mean ± SD (%)** |  | **GP-Adolescents**  **Mean ± SD (%)** | | | |
| --- | --- | --- | --- | --- | --- | --- | --- | --- | --- | --- | --- |
|  | **Males** | | **Females** | |  | **Females** |  | **Males** | | **Females** | |
|  | **Baseline**  (N = 55) | **Follow-up**  (N = 53) | **Baseline**  (N = 76) | **Follow-up**  (N = 72) |  | (N = 927) |  | **Baseline**  (N = 738) | **Follow-up**  (N =590) | **Baseline**  (N = 949) | **Follow-up**  (N = 846) |
| **AUDIT** | 10.7 ± 8.1 (96.4) | 12 ± 7.4 (94.3) | 10.8 ± 8.3 (92.1) | 8.6 ± 6.3 (91.7) | **AUDIT** | 6 ± 4.3 (92) | **AUDIT-C** | 0.3 ± 1.2 (10.2) | 3.3 ± 3.4 (61.9) | 0.5 ± 1.5 (13.3) | 3.2 ± 3.2 (63.1) |
| **SLE (types)**  None  One  Two  Three | 32.7%  40%  21.8%  5.5% | 37.7%  41.5%  20.8%  0% | 22.3%  31.6%  31.6%  14.5% | 23.6%  30.6%  26.4%  19.4% | **SLE (types)**  None  One  Two  Three  Four  Five | 34.9%  27.2%  16.9%  11.4%  7%  2.6% | **SLE** | 0.7 ± 1.4 (29.3) | 1.1 ± 1.7 (43.9) | 0.8 ± 1.8 (29.6) | 1.5 ± 2.1 (50.4) |
| **Child-parent openness**  **Parent-child affect**  **Parent-child support** | 12 ± 5.8  12.6 ± 4.6  11 ± 3.3 | na | 10.9 ± 5.7  11.2 ± 6.7  9.7 ± 4 | na | **Parent-child relationship** | 4.3 ± 1.7 | **PASCQ positive** | na | 28 ± 5.3 | na | 28.5 ± 5.3 |
| **Nicotine use** | 79.6% | 88.7% | 82.2% | 80% | **Nicotine use** | 52.9% | **Nicotine use** | 4.7% | 23.7% | 6.1% | 20.4% |

AUDIT: Alcohol Use Disorders Identification Test; AUDIT-C: AUDIT-Consumption; na: not assessed; ns: non-significant; PASCQ: Parents as Social Context Questionnaire

**CS:** Weak correlations were observed between AUDIT scores and SLE in both sexes (baseline: males: *r*: 0.376, *p* = 0.005; females: *r*: 0.218, *p* = 0.059; follow-up: males: *r*: 0.306, *p* = 0.026; females: *r*: 0.344, *p* = 0.003). At baseline, in the whole sample, AUDIT scores were negatively correlated with child-parent openness (*r* = -0.194; *p* = 0.028) and parent-child support (*r* = -0.283; *p* = 0.001). At follow-up, in the whole sample, AUDIT scores were negatively correlated with parent-child affect (*r* = -0.184; *p* = 0.043). Baseline AUDIT scores were negatively correlated with parent-child support in males (*r =* -0.480; *p* = 0.0002), while follow-up AUDIT scores were negatively correlated with child-parent openness in females (*r =* -0.317; *p* = 0.007). SLE at baseline was negatively correlated with parent-child affect (*r* = -0.308, *p* = 0.0004) and parent-child support (*r* = -0.252, *p* = 0.004) in the whole sample. In males the correlation was seen only with affect (*r* = -0.386, *p* = 0.004); and in females with both affect (*r* = -0.250, *p* = 0.029) and support (*r* = -0.233, *p* = 0.043). SLE at follow-up was negatively correlated with parent-child affect (*r* = -0.287, *p* = 0.001) and parent-child support (*r* = -0.187, *p* = 0.038) in the whole sample. In females, only the correlation with parent-child affect was significant (*r* = -0.300, *p* = 0.011). Carriers of the minor allele (T) reported lower AUDIT scores at baseline (*U* = 1331.5, *p* = 0.011) compared to CC individuals, especially in females (*U* = 389.5, *p* = 0.009). Males reported higher AUDIT scores at follow-up (*U* = 1400, *p* = 0.011) compared to females. Smokers reported higher AUDIT scores at baseline (*U* = 871, *p* = 0.024) compared to non-smokers. No nicotine use differences between the sexes were found. At baseline, but not at follow-up, CC were using more nicotine than T carriers (χ^2^ = 13.184; *p* = 0.001), especially males (χ^2^ = 10.895; *p* = 0.002).

**GP-Adults:** A weak correlation was found between AUDIT scores and SLE (*r* = 0.097; *p* = 0.003) but not with parent-child relationship (*p >* 0.05). SLE was negatively correlated with parent-child relationship (*r*: -0.267; *p* < 0.0001). There was no difference in AUDIT scores of T and CC carriers (*p >* 0.05). Nicotine use was associated with higher AUDIT scores (*U* = 64389; *p* < 0.0001). Genotype frequencies did not differ between participants using or not using nicotine.

**GP-Adolescents:** AUDIT-C scores were positively correlated with SLE scores at baseline (males: *r* = 0.189, *p* < 0.0001; females: *r* = 0.199, *p* < 0.0001), and negatively correlated with parenting style scores at follow-up (*r* = -0.112, *p* = 0.00002), especially in females (*r* = -0.140, *p* = 0.00005). Female carriers of the minor allele (T) reported lower AUDIT-C scores at follow-up compared to CC carriers (*U* = 70094, *p* = 0.028). At follow-up, females reported higher SLE scores than males (*U* = 224110, *p* = 0.001), and their AUDIT-C scores were positively correlated with SLE (*r* = 0.133, *p* < 0.0001). SLE and parenting at follow-up were negatively correlated (males: *r* = -0.197, *p* < 0.0001; females: *r* = -0.416, *p* < 0.0001). Nicotine use was associated with higher AUDIT-C scores at baseline (males: *U* = 3345, *p* < 0.0001; females: *U* = 6799, *p* < 0.0001) and follow-up (males: *U* = 11424.5, *p* < 0.0001; females: *U* = 16155, *p* < 0.0001). No sex differences in nicotine use were found. At follow-up, but not at baseline, CC were using more nicotine than T carriers (χ^2^ = 4.887; *p* = 0.027).

**Table S4**

Example questions, possible answers and sensitivity / specificity values of the environmental measures

| Environmental measure, example question | Possible answers | Reliability (Cronbach’s alpha) |
| --- | --- | --- |
| Conflict Tactics Scale Parent Child Version   - Mom/Dad has hit me with the fist or kick me hard? | 0: No/never; 1: Yes | 0.80 |
| Sexual Experience Survey questionnaire   - Have you given into sex play (fondling, kissing, or petting, but not intercourse) when you didn't want to because you were overwhelmed by a man's continual arguments and pressure? | 0: No; 1: Yes | 0.71 |
| Sexual and Physical Abuse Questionnaire   - Has anyone ever touched your sex organs in a sexual manner and against your will? | 0: No; 1: Yes | 0.82 |
| Victimization by peers   - Have you been exposed to others attacked you for no reason any weekday evening or on weekends? | 0: No; 1: Yes | 0.66 |
| Victimization by others:  McArthur Community Violence Instrument   - Has anyone thrown something at you? | 0: No; 1: Yes | 0.82 |
| Parent-child openness   - If you are worried about something, you talk to any of your parent / parents about it? | 0 - 4: Never to very often/always | 0.88 |
| Parent-child affection   - Do your parents display with words and gestures that they like you? | 0 - 4: Never to very often/always | 0.88 |
| Parent-child support   - When I am angry, sad or worried my mom / dad make me feel better | 0 - 6: “Not agree at all” to “totally agree” | 0.89 |
| Physical violence   - Has anyone ever hit or attacked you on purpose with an object or weapon? | 0 – 5: No to 5 times or more | 0.86[[2](#_ENREF_2)] |
| Verbal aggression   - Have you ever gotten scared or felt really bad because grown-ups in your life called you names, said mean things to you, or said that they didn’t want you? | 0 – 5: No to 5 times or more | 0.58 [[2](#_ENREF_2)] |
| Sexual abuse   - Has anyone ever touched your private parts when you didn’t want it or make you touch their private parts or forced you to have sex? | 0 – 5: No to 5 times or more | 0.84 [[2](#_ENREF_2)] |
| Neglect   - When you were a child did you ever have serious concerns, or were you ever sad or worried without having anyone that could help you, listen to you, comfort you and take your concerns seriously and protect you against threats? | 0 – 5: No to 5 times or more | 0.77 [[2](#_ENREF_2)] |
| Witnessing violence   - Have you ever seen one of your parents get hit, slapped, punched, or beat up? | 0 – 5: No to 5 times or more | 0.63 [[2](#_ENREF_2)] |
| Parent-child relationship   - When you were growing up, how often showed your parents clearly that they thought about you, such as by saying it or give you a hug or kiss? | 0 – 3: Never to very often | 0.66 |
| Family maltreatment   - Has it happened that one of your parents push, hit or use any other form of violence against you? | 0 – 5: Never to every/almost every day | 0.54 |
| PASCQ Positive  Warmth   - My parents show that they love me | 0 – 3: “Not agree at all” to “totally agree” | 0.81 |
| PASCQ Positive  Structure   - When I want to do something, my parents show me how | 0 – 3: “Not agree at all” to “totally agree” | 0.64 |
| PASCQ Positive  Autonomy-support   - My parents let me do the things I think are important | 0 – 3: “Not agree at all” to “totally agree” | 0.74 |

*In silico* search

The potential functional role of rs2290045 was investigated using bioinformatic tools (May 2018). Using RegulomeDB database[[3](#_ENREF_3)] (<http://www.regulomedb.org/>), a score of 6 for rs2290045 indicated no evidence of a functional role. Furthermore, using the SNAP server [[4](#_ENREF_4)] to identify the SNPs in LD with rs2290045 with r^2^ > 0.2 (<http://archive.broadinstitute.org/mpg/snap/ldsearch.php>), only one SNP (rs2665698) had a score of 5 showing evidence of minimal functionality, related to DNase sensitivity. Neither of the two SNPs are expression quantitative trait loci (eQTLs) for any gene in any tissue. The Genotype-Tissue Expression (GTEx) database was used to test the hypothesis that the SNP is not an eQTL (<http://www.gtexportal.org/home/testyourown>). In the hypothalamus, which is the tissue with the highest *VGLUT2* expression (<http://www.gtexportal.org/home/>; http://www.proteinatlas.org/), the rank normalized gene expression did not differ between homozygous for the major allele and heterozygotes (the TT group had only 1 individual). However, when choosing the hippocampus, a difference was observed between the CC and the TC groups, with the last showing lower gene expression (*p* = 0.02). Only two individuals were present in the TT group; due to the small number of available samples, tissue specific expression analysis in GTEx is likely underpowered to detect small (but real) effects.

**References**

1. Keller, M.C., *Gene x environment interaction studies have not properly controlled for potential confounders: the problem and the (simple) solution.* Biol Psychiatry, 2014. **75**(1): p. 18-24.

2. Cater, A.K., A.K. Andershed, and H. Andershed, *Youth victimization in Sweden: prevalence, characteristics and relation to mental health and behavioral problems in young adulthood.* Child Abuse Negl, 2014. **38**(8): p. 1290-302.

3. Boyle, A.P., et al., *Annotation of functional variation in personal genomes using RegulomeDB.* Genome Res, 2012. **22**(9): p. 1790-7.

4. Johnson, A.D., et al., *SNAP: a web-based tool for identification and annotation of proxy SNPs using HapMap.* Bioinformatics, 2008. **24**(24): p. 2938-9.
